# Acute respiratory distress syndrome caused by salicylate intoxication

**DOI:** 10.1002/ccr3.1729

**Published:** 2018-07-23

**Authors:** Yuichiro Otani, Keishi Kanno, Ezekiel Wong Toh Yoon, Susumu Tazuma

**Affiliations:** ^1^ Department of General Internal Medicine Hiroshima University Hospital Minami‐ku, Hiroshima Japan; ^2^ Department of Internal Medicine Hiroshima Kyouritsu Hospital Hiroshima Japan

**Keywords:** acute respiratory distress syndrome, intoxication, metabolic acidosis, salicylate

## Abstract

Salicylate‐induced acute respiratory syndrome (ARDS) is a well‐known entity occurring in 35% of salicylate‐intoxicated patient. Careful history taking, physical examination, arterial blood gas analysis, and measurement of serum salicylate concentration will lead to early recognition to initiate appropriate treatment.

A 65‐year‐old previously healthy female was admitted because of altered mental status and respiratory failure. Owing to lumbago, she had been taking acetylsalicylate tablets for 2 weeks (total amount, 52.8 g). The physical examination findings were as follows: body temperature, 36.1°C; blood pressure, 88/54 mm Hg, pulse, 89 beats/min. In addition, she was hyperpneic with the respiratory rate being 32 breaths/min and peripheral oxygen saturation being 92% while breathing 5 L of oxygen per minute. The blood gas analysis indicated metabolic acidosis with anion gap elevation and respiratory alkalosis. While echocardiography upon admission was unremarkable, chest radiograph and CT of the lung revealed massive bilateral ground‐glass appearance (Figure 1A,B). The blood glucose level was 151 mg/dL, and brain CT was unremarkable. Standard microbiological screening did not reveal any infective organism. Based on the patient's medical history and findings of clinical examinations, we speculated the probable diagnosis of salicylate intoxication 1, 2. Few days after admission, a significantly high level of salicylate concentration was reported in the serum obtained on the day of admission [720 g/mL (reference range, 100‐250 g/mL)], leading to the diagnosis of ARDS due to salicylate intoxication.

**Figure 1:**
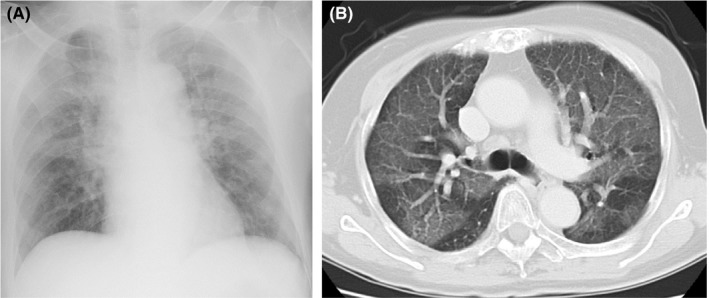
A, B, Chest x‐ray and CT perfomed on admission showed bilateral ground glass opacity dominant on the upper lung field

## CONFLICT OF INTEREST

None declared.

## AUTHORSHIP

YO: contributed to treat the patient and drafted the manuscript, KK: revised the manuscript and contributed as a corresponding author, EWTY: contributed to treat the patient, and ST: critically reviewed the literature and involved in writing. All authors approved the final manuscript.
